# The obesity paradox and hypoglycemia in critically ill patients

**DOI:** 10.1186/s13054-021-03795-z

**Published:** 2021-11-01

**Authors:** Drago Plečko, Nicolas Bennett, Johan Mårtensson, Rinaldo Bellomo

**Affiliations:** 1 Seminar for Statistics, Department of Mathematics, ETH Zürich, Zürich, Switzerland; 2 Department of Perioperative Medicine and Intensive Care, Karolinska University Hospital, Stockholm, Sweden; 3 Department of Physiology and Pharmacology, Section of Anaesthesia and Intensive Care, Karolinska Institutet, Stockholm, Sweden; 4 Australian and New Zealand Intensive Care Research Centre, School of Public Health and Preventative Medicine, Monash University, Melbourne, Australia; 5 Department of Critical Care, The University of Melbourne, Melbourne, Australia; 6 Data Analytics Research and Evaluation Centre, Department of Medicine and Radiology, The University of Melbourne, Melbourne, Australia; 7 Austin Hospital, Melbourne, Australia

**Keywords:** Obesity, Overweight, Hypoglycemia, Glucose, Insulin, Mortality, Outcome, Intensive care, Critical illness

## Abstract

**Background:**

A high body mass index (BMI) has been associated with decreased mortality in critically ill patients. This association may, in part, relate to the impact of BMI on glycemia. We aimed to study the relationship between BMI, glycemia and hospital mortality.

**Methods:**

We included all patients with a recorded BMI from four large international clinical databases (*n* = 259,177). We investigated the unadjusted association of BMI with average glucose levels, mortality and hypoglycemia rate. We applied multivariate analysis to investigate the impact of BMI on hypoglycemia rate, after adjusting for glycemia-relevant treatments (insulin, dextrose, corticosteroids, enteral and parenteral nutrition) and key physiological parameters (previous blood glucose level, blood lactate, shock state, SOFA score).

**Results:**

We analyzed 5,544,366 glucose measurements. On unadjusted analysis, increasing BMI was associated with increasing glucose levels (average increase of 5 and 10 mg/dL for the 25–30, 30–35 kg/m^2^ BMI groups compared to normal BMI (18.5–25 kg/m^2^) patients). Despite greater hyperglycemia, increasing BMI was associated with lower hospital mortality (average decrease of 2% and 3.25% for the 25–30, 30–35 kg/m^2^ groups compared to normal BMI patients) and lower hypoglycemia rate (average decrease of 2.5% and 3.5% for the 25–30, 30–35 kg/m^2^ groups compared to normal BMI patients). Increasing BMI was significantly independently associated with reduced hypoglycemia rate, with odds ratio (OR) 0.72 and 0.65, respectively (95% CIs 0.67–0.77 and 0.60–0.71, both *p* < 0.001) when compared with normal BMI. Low BMI patients showed greater hypoglycemia rate, with OR 1.6 (CI 1.43–1.79, *p* < 0.001). The association of high BMI and decreased mortality did not apply to diabetic patients. Although diabetic patients had higher rates of hypoglycemia overall and higher glucose variability (*p* < 0.001), they also had a reduced risk of hypoglycemia with higher BMI levels (*p* < 0.001).

**Conclusions:**

Increasing BMI is independently associated with decreased risk of hypoglycemia. It is also associated with increasing hyperglycemia and yet with lower mortality. Lower risk of hypoglycemia might contribute to decreased mortality and might partly explain the obesity paradox. These associations, however, were markedly modified by the presence of diabetes.

**Graphical Abstract:**

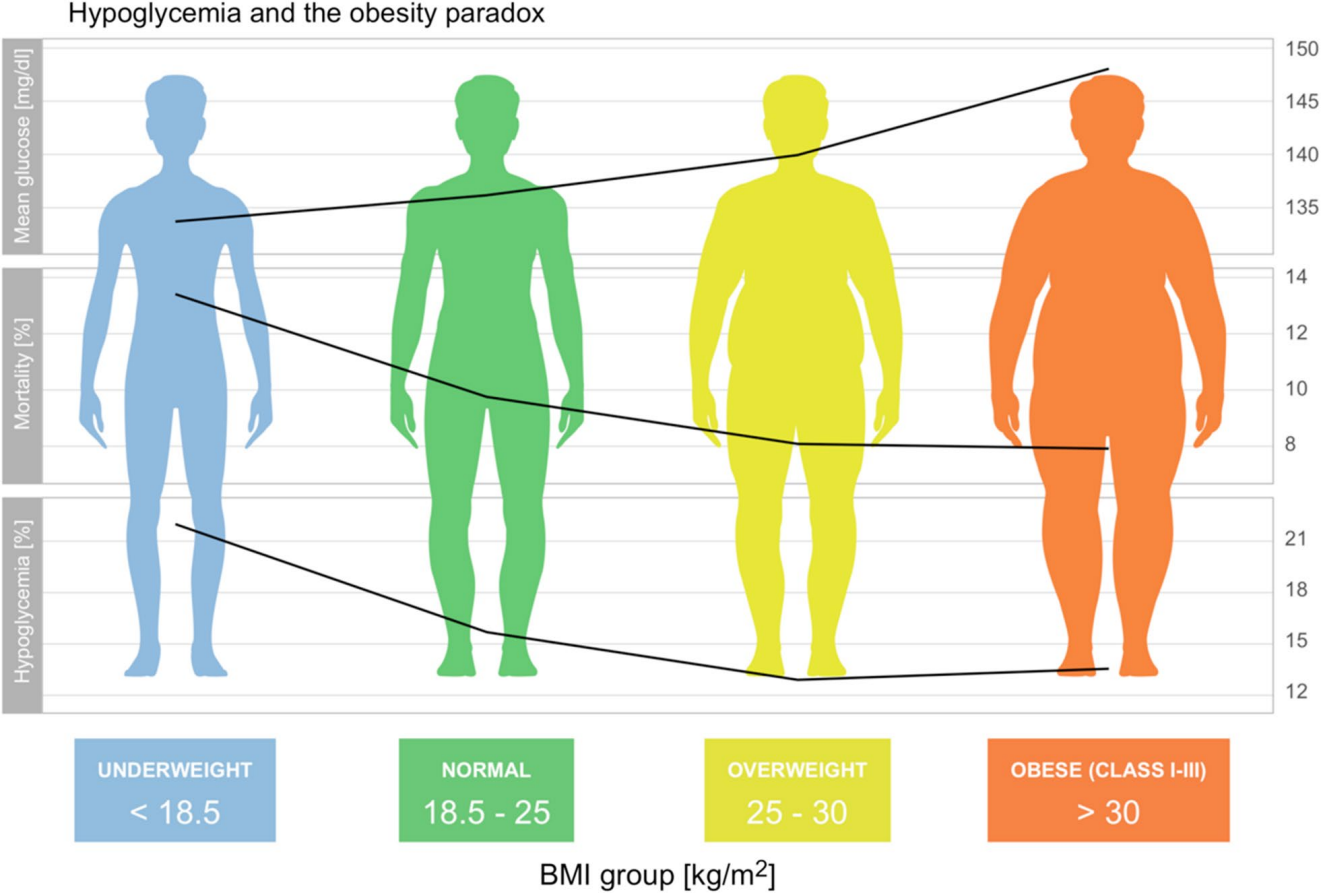

**Supplementary Information:**

The online version contains supplementary material available at 10.1186/s13054-021-03795-z.

## Introduction

Overweight and obesity are associated with numerous comorbidities and risk factors for life-threatening complications [1–3]. They are also strongly associated with increased long-term mortality [4, 5]. In contrast, multiple studies have found that overweight and mildly obese patients have lower short-term mortality rates than their leaner counterparts, after both cardiac [6–11] and non-cardiac [12–17] surgery. Furthermore, a similar phenomenon has been observed in non-surgical populations [18–20]. This counterintuitive relationship between overweight and short-term mortality has been termed ‘the obesity paradox’ [6–17] and a definite explanation for it has not yet been established. A possible partial explanation for this paradox, however, might relate to glycemic control.

The role of fat tissue in modulating glucose homeostasis is well-established [21, 22]. The most commonly used surrogate for measuring excess fat tissue is the body-mass index (BMI). In this regard, using a national health database, an investigation of the relationship between BMI and severe hypoglycemia in more than one million Korean ambulant type 2 diabetic patients [23] recently found that BMI and hypoglycemia were inversely associated. Thus, a higher BMI appears to protect against hypoglycemia. However, the association of BMI and hypoglycemia in intensive care patients remains unexplored.

Accordingly, we aimed to explore the association of BMI with mortality and glycemia in patients from a group of large intensive care datasets. In particular, we aimed to investigate the hypothesis that patients with increased BMI have an increased rate of hyperglycemia, but a decreased rate of both hypoglycemia and mortality. Finally, we sought to understand how these associations are affected by the diagnosis of diabetes.

## Methods

### Study cohort

We studied patients from four large intensive care electronic health records (EHR) databases: (a) the Amsterdam University Medical Center Database (AUMC) [24] from the Amsterdam University Medical Center, collected between 2003 and 2016; (b) the High Time Resolution Intensive Care Database (HiRID) [25] from the Department of Intensive Care Medicine of the University Hospital of Bern, collected between 2008 and 2016; (c) the Medical Information Mart for Intensive Care (MIMIC-III) [26] database from the Beth Israel Deaconess Medical Center in Boston, Massachusetts, collected between 2001 and 2012; and (d) the eICU Collaborative Research Database [27], containing data collected from 206 hospitals across the USA in 2014 and 2015.

**Fig. 1:**
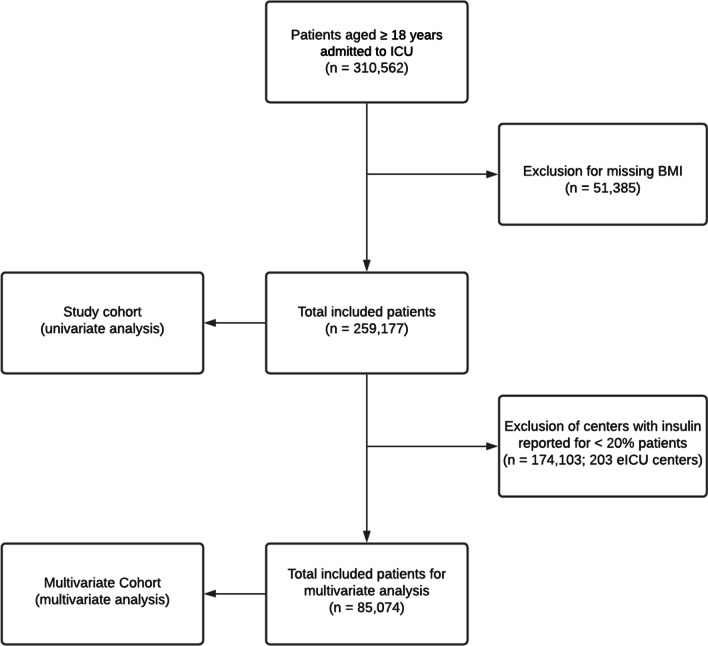
Study flowchart with patient inclusion/exclusion criteria

All adult patients (≥ 18 years) with recorded height and weight at time of ICU admission from the four databases were included in the analysis. The body-mass index (BMI) was calculated using height and weight at admission and variations in BMI during stay were not considered in the analyses. For a secondary univariate analysis using HbA1c levels, a subset of the MIMIC-III cohort with recorded HbA1c values was used. In order to decrease potential underreporting bias for insulin, for multivariate analyses all hospitals in the eICU database where < 20% of patients received insulin therapy were excluded from analysis (the threshold was chosen to match the insulin prevalence in the lowest prevalence MIMIC-III ICU). We refer to the resulting cohort as the Multivariate Cohort. The study flowchart of patients is shown in Fig. 1.

The primary outcome was occurrence of hypoglycemia (blood glucose ≤ 70 mg/dL [≤ 3.9 mmol/L]). The secondary outcome was in-hospital mortality. However, for the HiRID dataset, in-hospital mortality information was not reported and therefore ICU mortality was used on this dataset. The blood glucose target range in the HiRID database was 81–180 mg/dL (4.5–10 mmol/L). In the AUMC database, the target value was 117 mg/dL (6.5 mmol/L) for patients monitored using the Glucose Regulation for Intensive Care Patients (GRIP) system [28], whereas the target range was 110–160 mg/dL in the guidelines for non-computerized monitoring, used during the last year of data collection.

We obtained data on the following potentially important physiological and treatment factors for development of hypoglycemia: (a) prior glucose levels, (b) lactate levels, (c) presence or absence of shock (defined as mean arterial pressure < 60 mmHg or administration of vasopressors), (d) administered insulin dose (the maximum hourly infusion rate in U/h in the 12 h preceding an event, with intermittent bolus administrations included where available), (e) administered dextrose dose (hourly infusion rate in mL/h normalized to equivalents of dextrose 10%; dextrose 5% was not included), (f) presence of parenteral nutrition, (g) presence of enteral nutrition and (h) presence of corticosteroid administration. We also estimated the hourly Sequential Organ Failure Assessment (SOFA) scores [29] using the relevant values for each component in all patients. We reported the missingness of key variables considered at 24 h into ICU stay.

All the data used in our analyses are publicly available. The AUMC Database is available through the website of Amsterdam Medical Data Science [30]. HiRID, MIMIC-III and eICU databases are available through PhysioNet [31].

### Univariate analyses

For the univariate analyses, we first investigated whether the BMI had an association with average glucose levels. We did this by categorizing patients into BMI groups according to the World Health Organization classification: underweight (0–18.5 kg/m^2^), normal (18.5–25 kg/m^2^), overweight (25–30 kg/m^2^), class I obese (30–35 kg/m^2^), class II obese (35–40 kg/m^2^) and class III obese (> 40 kg/m^2^). For each patient, we established the time-weighted average glucose level during ICU stay (times after first hypoglycemia onset not included). Then, for each group, we calculated the mean of the time-weighted average glucose values. Further, we determined the mortality rate for each BMI group and the proportion of patients who developed an episode of hypoglycemia during ICU stay. We also determined the average coefficient of variation of the blood glucose levels in each group. These analyses were then repeated by stratifying on type of admission, to determine whether there were important differences between admission types.

Further, we repeated the analyses by stratifying according to diagnosis of diabetes (here only the US databases were used, where the diabetes diagnosis was reported using ICD-9 codes), to understand the impact of diabetes on the association of BMI with glycemic outcomes and mortality. Moreover, in a secondary analysis of the impact of diabetes, using a subset of the MIMIC-III database where HbA1c levels were recorded, we stratified patients according to HbA1c levels into four bins: non-diabetic (< 6.1%), pre-diabetic (6.1–6.5%), moderately controlled diabetes (6.6–7.0%), poorly controlled diabetes (> 7.0%).

To investigate the association of glycemic outcomes and mortality and how they changed according to BMI and diabetes status, we performed the following analyses. For time-weighted average glucose, we grouped patients into three bins: 70–140, 140–180, > 180 mg/dL. For each bin of time-weighted average glucose, we then assessed the mortality rate according to BMI group and diagnosis of diabetes (the analysis was repeated for the subset of patients who did not develop hypoglycemia during ICU stay). A similar analysis was performed for the blood glucose coefficient of variation, using four bins (< 10%, 10–20%, 20–30%, > 30%) and hypoglycemia, using three bins (0, 1 or > 1 episode of hypoglycemia).

Additionally, we assessed several process of care characteristics. For each BMI group, we calculated the average hourly frequency of glucose measurements, within the same time window as described above. Using only the patients in the top quartile with respect to glucose measurement frequency, we again computed the proportion of patients who experienced hypoglycemia in each BMI group, to determine whether the frequency of monitoring affected the findings. Moreover, we computed the mortality rate in each BMI group using only patients who developed hypoglycemia. In order to investigate whether the severity of hypoglycemia differed across BMI groups, for the hypoglycemic cohort, for each patient, we also calculated (1) the average of the lowest blood glucose measurement; (2) the overall hypoglycemic load (defined as the proportion of glucose measurements that were ≤ 70 mg/dL); (3) the number of hypoglycemic episodes (an episode is assumed to end 6 h after a hypoglycemic measurement, unless terminated early by a non-hypoglycemic measurement, or prolonged by a subsequent hypoglycemic measurement). Finally, when considering medication, we calculated the average maximal insulin dose (both unadjusted and adjusted by patient weight), average mean dextrose dose and the average duration of treatment with parenteral/enteral nutrition and corticosteroids during ICU stay (excluding times after onset of hypoglycemia). The 95% confidence intervals (CIs) of mean values obtained using 500 bootstrap repetitions were included for all point estimates. For each analysis we tested whether there was a difference in distribution of values for patients with BMI < 25 kg/m^2^ versus patients with BMI ≥ 25 kg/m^2^. A *χ*^2^- test was used for binary variables, whereas a Mann–Whitney *U*-test was used for continuous variables and we reported the obtained *p*-values.

### Multivariate analysis

After establishing the univariate relation between BMI and hypoglycemia, we investigated whether BMI was an independent predictor of hypoglycemia in a multivariate model using the key variables described above. We included the BMI categories into the multivariate logistic regression model and, for each patient, the relevant parameters at 6 h into ICU stay (prior glucose levels, lactate levels, presence or absence of shock, administered insulin dose, dextrose dose, usage of parenteral/enteral nutrition and corticosteroids). We then determined whether the patient had a hypoglycemic episode within the subsequent 6 h (between 6 and 12 h). If a hypoglycemic episode occurred, this outcome was labelled as *H* = 1 (for hypoglycemia) and no further data for that patient were considered for analysis thereafter (i.e., only data before the first hypoglycemic event was analyzed). In the absence of a hypoglycemic event between 6 and 12 h into ICU stay, the outcome was labelled as *H* = 0, we assessed the subsequent 6-h window (between 12 and 18 h) and repeated the process in an iterative way thereafter.

We selected a window duration of 6 h as short enough for accurate predictions, but long enough to allow possible preventative intervention in future clinical practice. We carried less frequently measured variables (liver function tests) forward for up to 48 h, unless a new measurement was available. For more frequently measured variables (glucose, lactate, mean arterial pressure) the carry-forward period was always ≤ 24 h. If no subsequent measurement fell into the carry forward period, median imputation was used up to the next observed value.

The above multivariate logistic regression model was fitted on all of the data jointly, where we included the database as an additional predictor. We reported the estimated odds ratios (ORs) and their confidence intervals. A *χ*^2^-test was used to test for the significance of BMI as a categorical variable and its *p*-value was reported. We also inspected the generalized variance-inflation factors (GVIF) [32] of the model, to test for multicollinearity. Additionally, to address the level of missingness, a sensitivity analysis was conducted on a subset of the data where the missingness of BMI and lactate levels was low (< 5% missing BMI, < 15% missing lactate). The multivariate model was also fitted on the US databases only, where the diagnosis of diabetes was included as a predictor.

The Ethical Commission of Canton Zürich waived the need for an ethical approval of the study (Request-2021–00,618). For statistical analysis and data loading, we used the ricu R-package [33] and R Statistical Software [34] Version 4.1.0. All the code used in the analyses is available on Github https://github.com/eth-mds/bmi.

Throughout the text, where the relevant values are also reported per database, we report the value for the AUMC database first, followed by values for HiRID, MIMIC-III and eICU, respectively, unless otherwise explicitly stated.

## Results

The proportion of adult patients with a recorded BMI was 83.5% (93.6%, 92.5%, 46.2%, 90.7% per database) and the study cohort comprised of 259,177 (21,624, 31,348, 24,623, 181,582 per database) patients which had 5,544,366 glucose measurements.

For the multivariate analysis, we used the Multivariate Cohort, which comprised of 85,074 patients. The multivariate analysis considered 507,977 lactate measurements, 7,397,330 MAP values, 1,840,259 h of vasopressor administration, 1,302,135 h of insulin administration, 56,700 h of dextrose administration, 215,463 h of parenteral nutrition, 760,157 h of enteral nutrition and 59,064 h of corticosteroid treatment. For the secondary analysis investigating HbA1c levels, we used a subset of 4202 patients from the MIMIC-III cohort where there was a recorded value.

MIMIC-III patients had the longest average length of ICU and hospital stay. The average age was approximately 65 years across all cohorts with a slightly higher proportion of men (Table 1). SOFA scores and mortality rates in the cohorts are also shown in Table 1. Comparison of characteristics of patients with a recorded BMI and patients with a missing BMI is reported in Additional file 1.

**Table 1:** Patient characteristics and outcomes.

Variable	Reported	MIMIC-III	eICU	HiRID	AUMC
Cohort size	*n*	24,623	181,582	31,348	21,624
*Admission type*	%				
Medical Surgical Other		63 36 1	82 16 2	NR NR NR	22 71 7
Age (years)	Median (IQR)	66 (54–77)	65 (53–76)	65 (55–75)	65 (55–75)
Mortality	%	10.4	8.9	5.7*	9.8
Hospital LOS (days)	Median (IQR)	8.09 (5.01–14.48)	5.60 (2.96–10.12)	NR	NR
ICU LOS	Median (IQR)	2.78 (1.39–5.73)	1.69 (0.89–3.11)	1.00 (0.82–2.26)	1.08 (0.85–3.75)
Gender (male)	%	60	54	65	66
*BMI group* (kg/m^2^)	%				
[0–18.5] [18.5–25] [25–30] [30–35] [35–40] > 40		3 31 34 18 7 6	4 30 29 18 9 9	2 44 37 13 4 1	7 48 26 14 4 1
*SOFA score*	Median (IQR)				
Cardiovascular CNS Coagulation Hepatic Renal Respiratory Total		1 (1–1) 0 (0–1) 0 (0–1) 0 (0–0) 0 (0–1) 1 (0–2) 4 (2–6)	1 (1–1) 0 (0–2) 0 (0–1) 0 (0–1) 0 (0–1) 1 (0–2) 3 (1–6)	1 (1–4) 0 (0–1) 0 (0–1) 0 (0–0) 0 (0–1) 3 (2–4) 6 (4–9)	3 (1–4) 0 (0–1) 0 (0–1) 0 (0–1) 0 (0–1) 2 (2–3) 7 (5–9)

BMI, body-mass index; CNS, central nervous system; IQR, interquartile range; LOS, length of stay; NR, not reported; SOFA, Sequential Organ Failure Assessment, evaluated at 24 h into ICU stay

*ICU mortality reported for HiRID dataset

Hypoglycemia occurred in 36,731 (14.1%) patients. Missingness of physiological markers at 24 h into ICU stay by BMI group and dataset is reported in Additional file 2 and Additional file 3.

### Univariate comparisons according to BMI

**Fig. 2:**
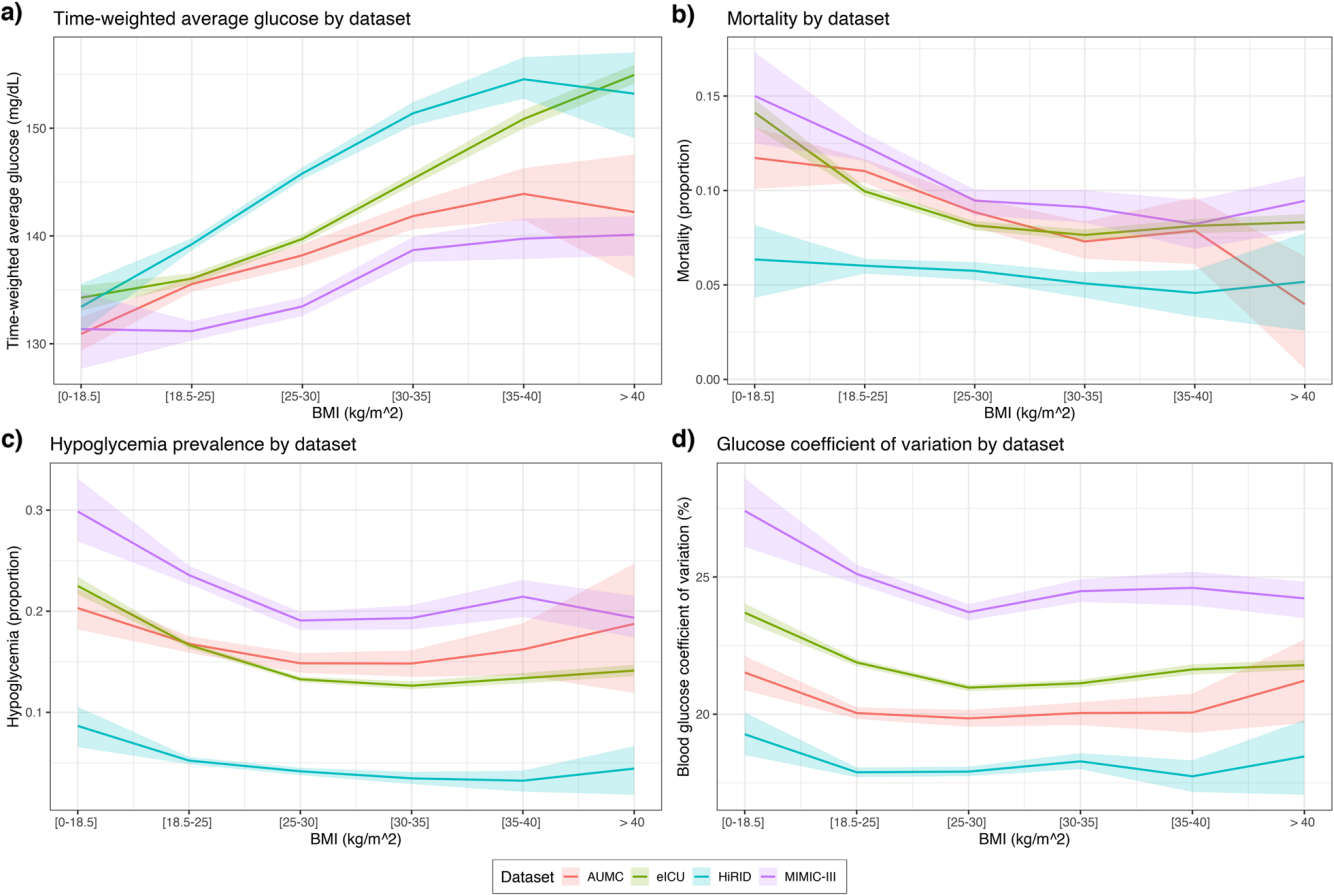
Mortality and key glycemic characteristics of each BMI group. **a** Increasing BMI values were associated with higher time-weighted glucose levels; **b** shows the obesity paradox in each dataset; **c** shows the rate of hypoglycemia; **d** shows the relationship of BMI with glycemic variability. Shaded regions around the curves present 95% confidence intervals of the point estimates, obtained using bootstrap

**Fig. 3:**
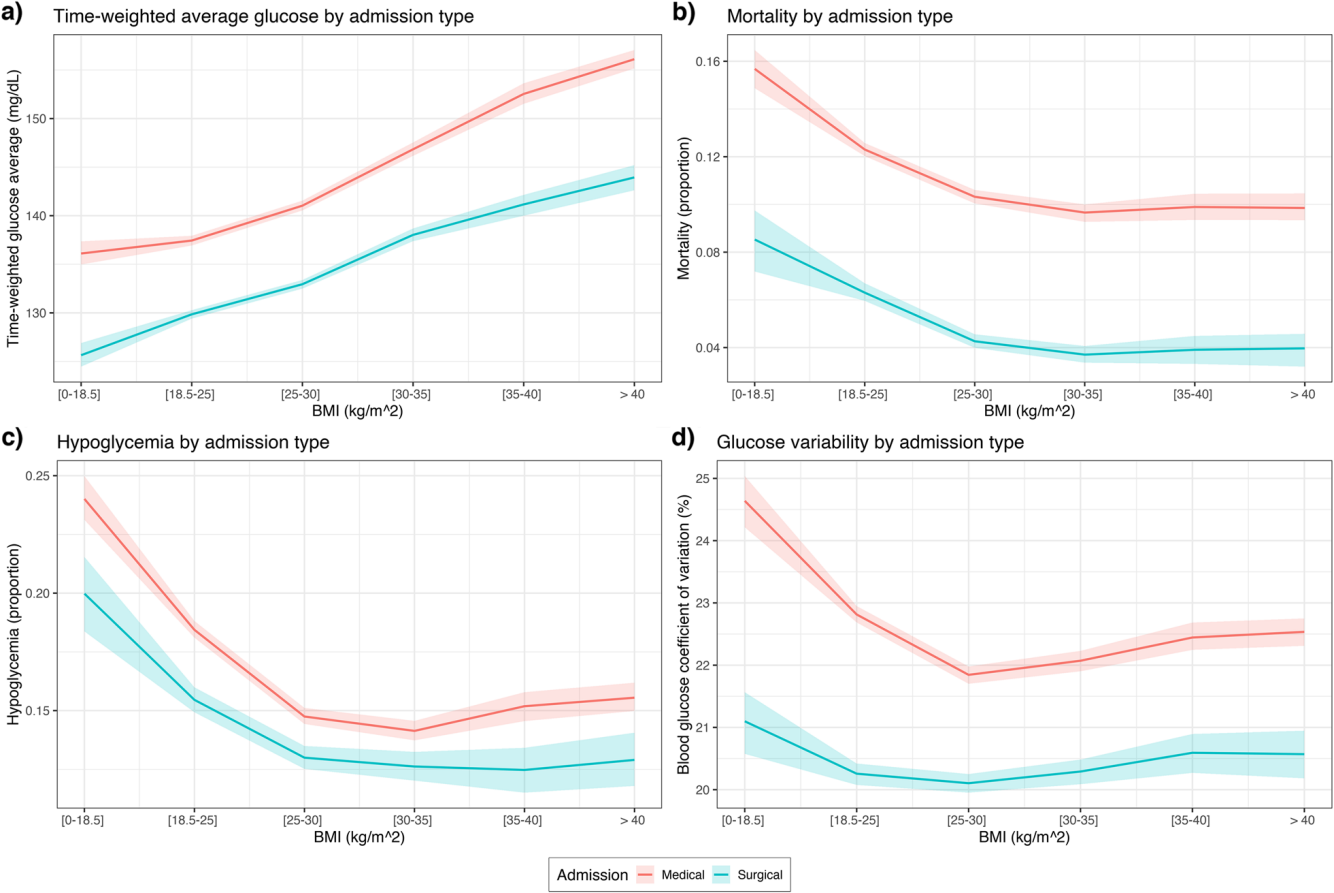
Mortality and key glycemic characteristics of each BMI group, conditional on admission type. Increasing BMI was associated with higher time-weighted average glucose (**a**), lower mortality (**b**), lower rate of hypoglycemia (**c**) and lower glucose variability (**d**), irrespective of admission type

On univariate analysis of the overall study population, increasing values of the BMI were associated with higher average glucose levels (Fig. 2a, all *p* < 0.001), lower mortality (Fig. 2b, *p* = 0.047 for HiRID, *p* < 0.001 for other), lower rate of hypoglycemia (Fig. 2c, all *p* < 0.001) and higher glucose variability (Fig. 2d, all *p* < 0.001). The same associations were observed irrespective of admission type (Fig. 3, all *p* < 0.001).

### Impact of diabetes

**Fig. 4:**
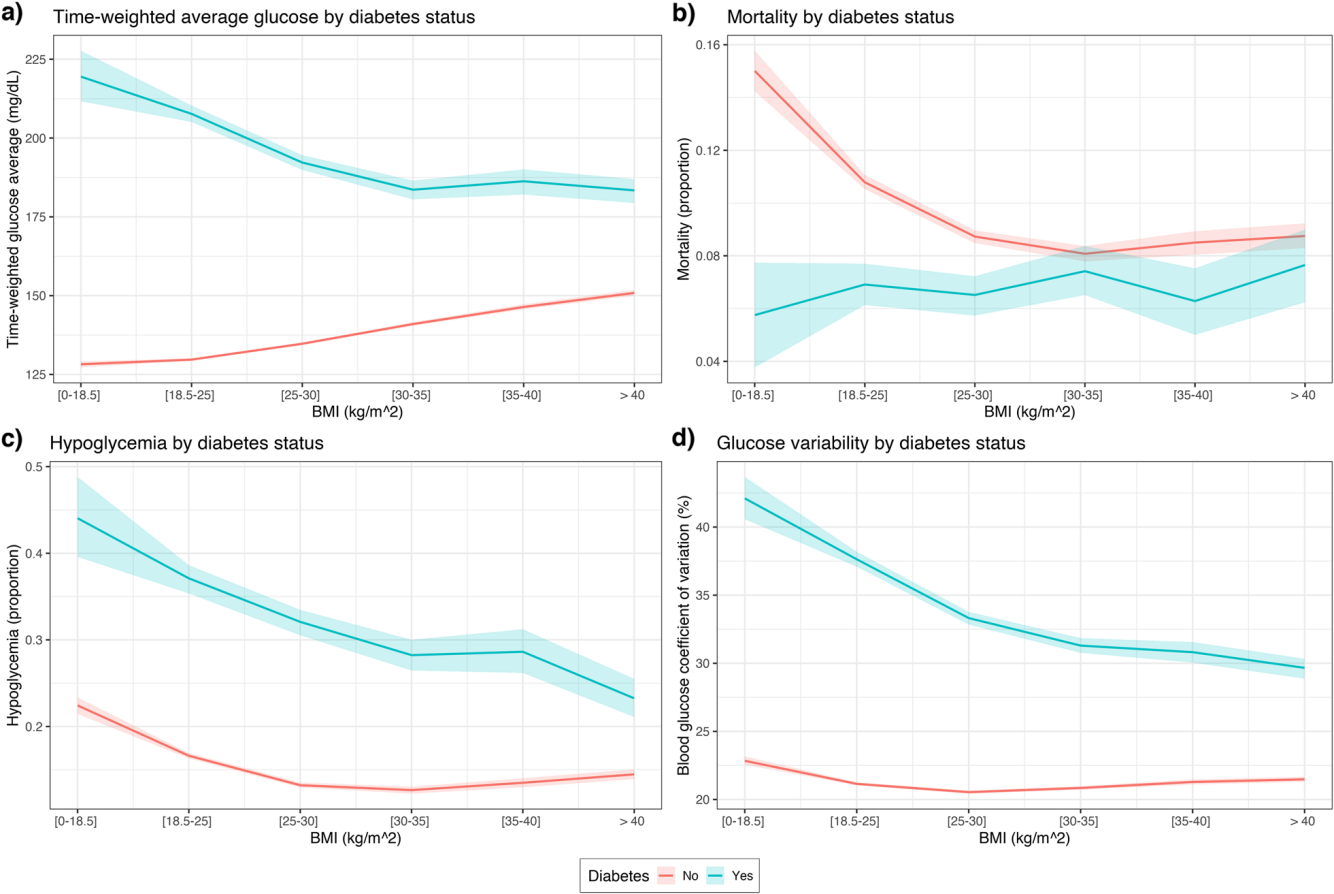
Mortality and key glycemic characteristics of each BMI group, conditional on diabetes status. Diabetes status modulated the relationship of BMI and glycemic outcomes and mortality. For diabetic patients, higher BMI was associated with lower average glucose (**a**), lower rate of hypoglycemia (**c**) and lower glucose variability (**d**). The null-hypothesis of no association of BMI with mortality for the diabetic group on the pooled dataset was not rejected at 5% significance level (**b**), although it was rejected when considering the MIMIC-III dataset only

**Fig. 5:**
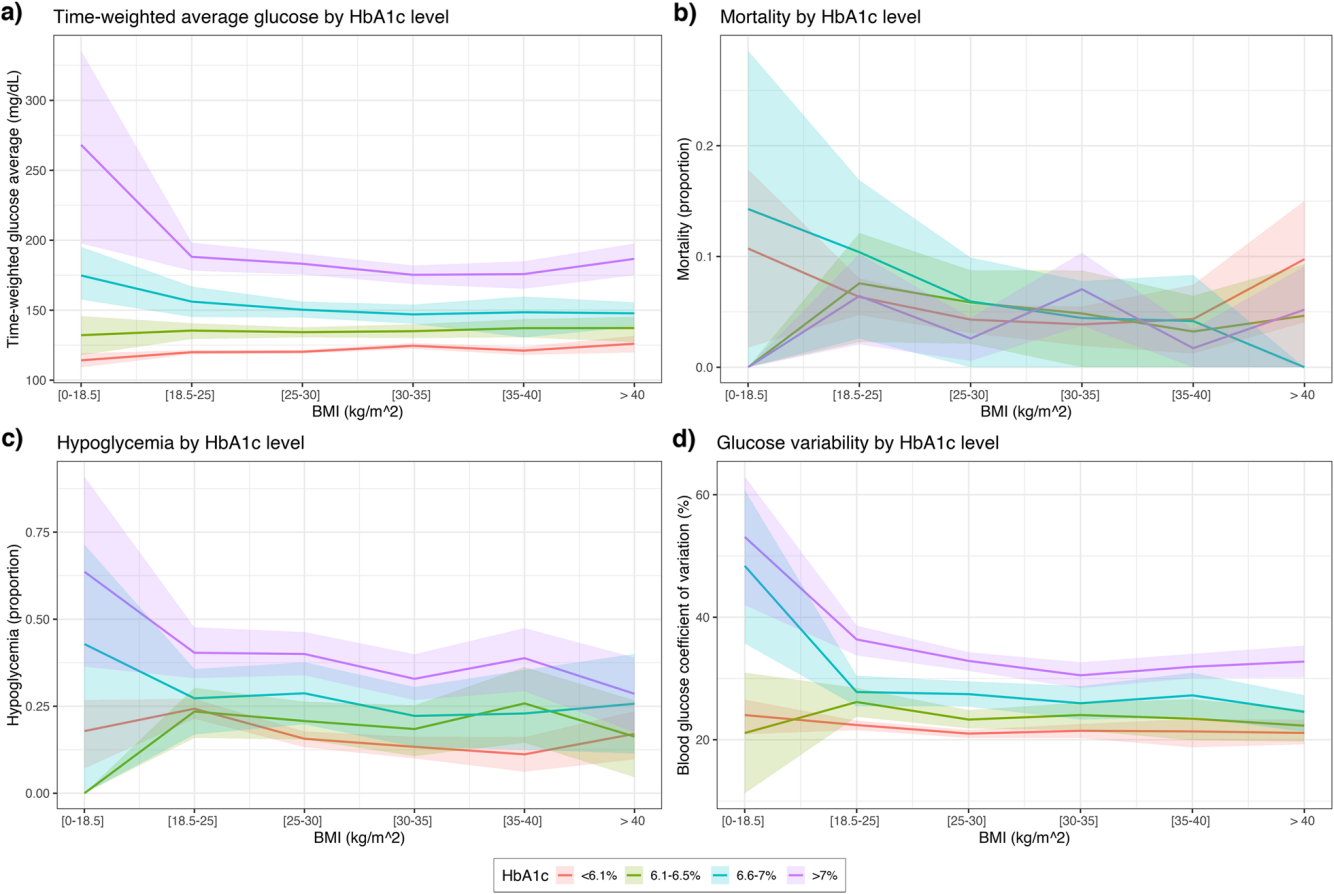
Mortality and key glycemic characteristics of each BMI group, conditional on HbA1c levels. HbA1c levels modulated the relationship of BMI and glycemic outcomes and mortality. For diabetic patients, higher BMI was associated with lower average glucose (**a**), lower rate of  hypoglycemia (**c**) and lower glucose variability (**d**). There was no clear association of BMI with mortality in the different HbA1c groups (**b**)

For diabetic patients, however, the association of time-weighted average glucose with BMI was reversed, meaning that low BMI patients had higher average glucose levels (Fig. 4a, *p* < 0.001). Furthermore, the association of high BMI and decreased mortality did not apply to diabetic patients in the pooled dataset (Fig. 4b, *p* = 0.81). However, on MIMIC-III dataset only, diabetic patients with higher BMI still had decreased mortality rates when compared to normal BMI patients (p < 0.001). Although diabetic patients had higher rates of hypoglycemia overall (Fig. 4c) and higher glucose variability (Fig. 4d, *p* < 0.001), they also had a reduced risk of hypoglycemia with higher BMI levels (Fig. 4d, *p* < 0.001). When considering HbA1c levels, we found for the < 6.1% group, increased BMI was associated with higher average glucose (*p* = 0.001), lower mortality (*p* = 0.04), and lower rate of hypoglycemia (*p* < 0.001), whereas for the other three groups the associations were not statistically significant (Fig. 5a–c, all *p* > 0.05). Increasing levels of BMI were associated with lower glucose variability in each HbA1c group (Fig. 5d, all *p* < 0.05).

### Association of glycemic outcomes with mortality

**Fig. 6:**
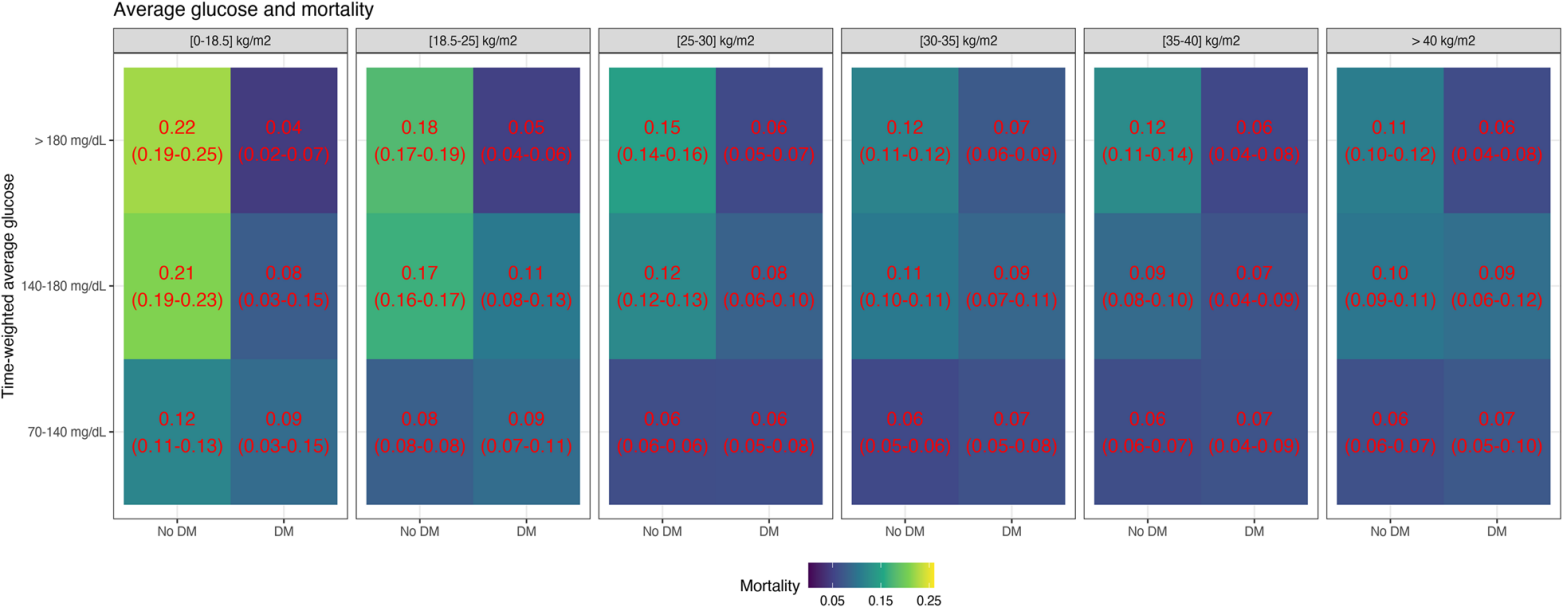
Mortality and time-weighted average glucose association conditional on BMI group and diabetes status. The figure shows the mortality rate in different bands of time-weighted average glucose, conditional on BMI group and diabetes status. For non-diabetic patients, there was an association of higher glucose levels and increased mortality, with the differences decreasing with BMI. For diabetic patients, higher glucose levels were not associated with increased mortality

**Fig. 7:**
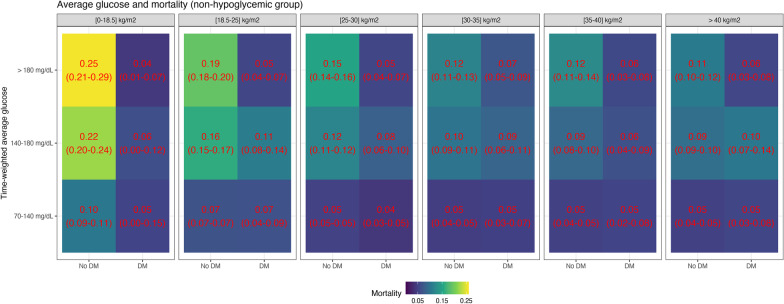
Mortality and time-weighted average glucose association conditional on BMI group and diabetes status for the non-hypoglycemic group. The figure shows the mortality rate in different bands of time-weighted average glucose, conditional on BMI group and diabetes status, for the subset of patients who did not develop hypoglycemia during ICU stay. For non-diabetic patients, there was an association of higher glucose levels with increased mortality, with the differences decreasing with BMI. For diabetic patients, higher glucose levels were not associated with increased mortality

**Fig. 8:**
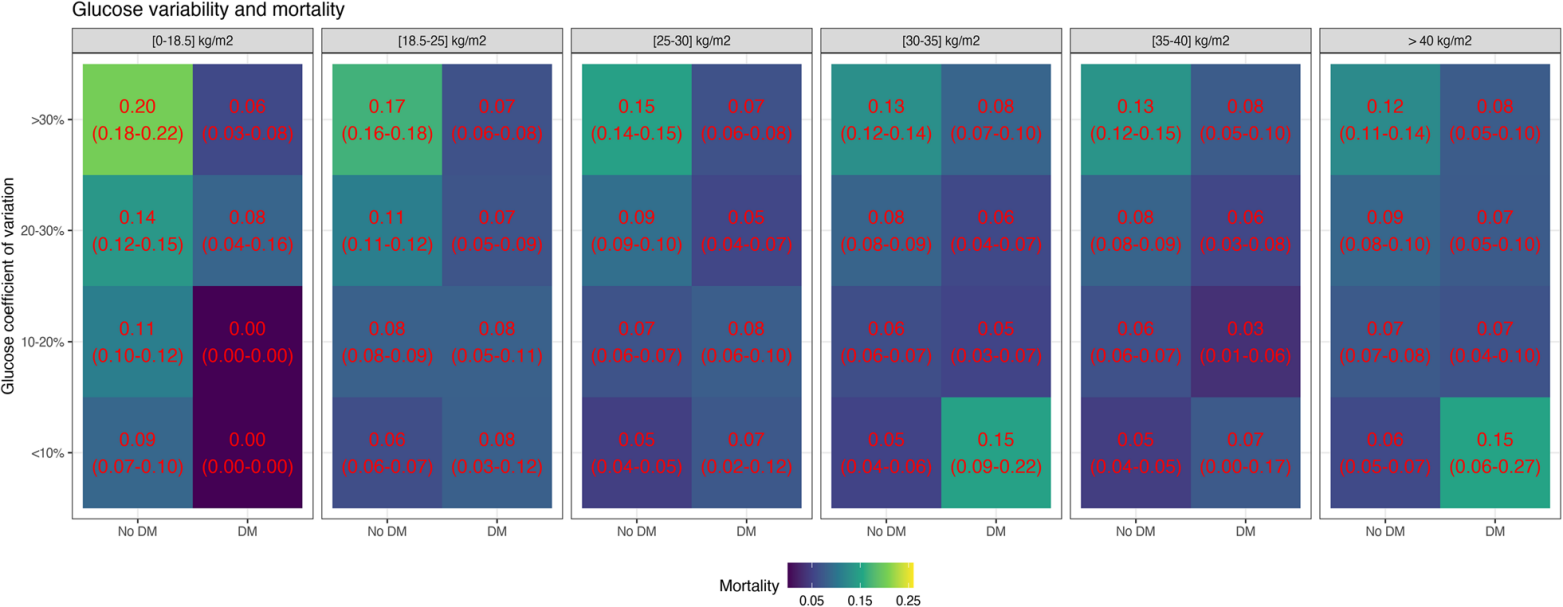
Mortality and glucose variability association conditional on BMI group and diabetes status. The figure shows the mortality rate in different bands of glucose coefficient of variation, conditional on BMI group and diabetes status. For non-diabetic patients, there was an association of higher glucose variability with increased mortality, with the differences decreasing with BMI. For diabetic patients, higher glucose variability was not associated with increased mortality

**Fig. 9:**
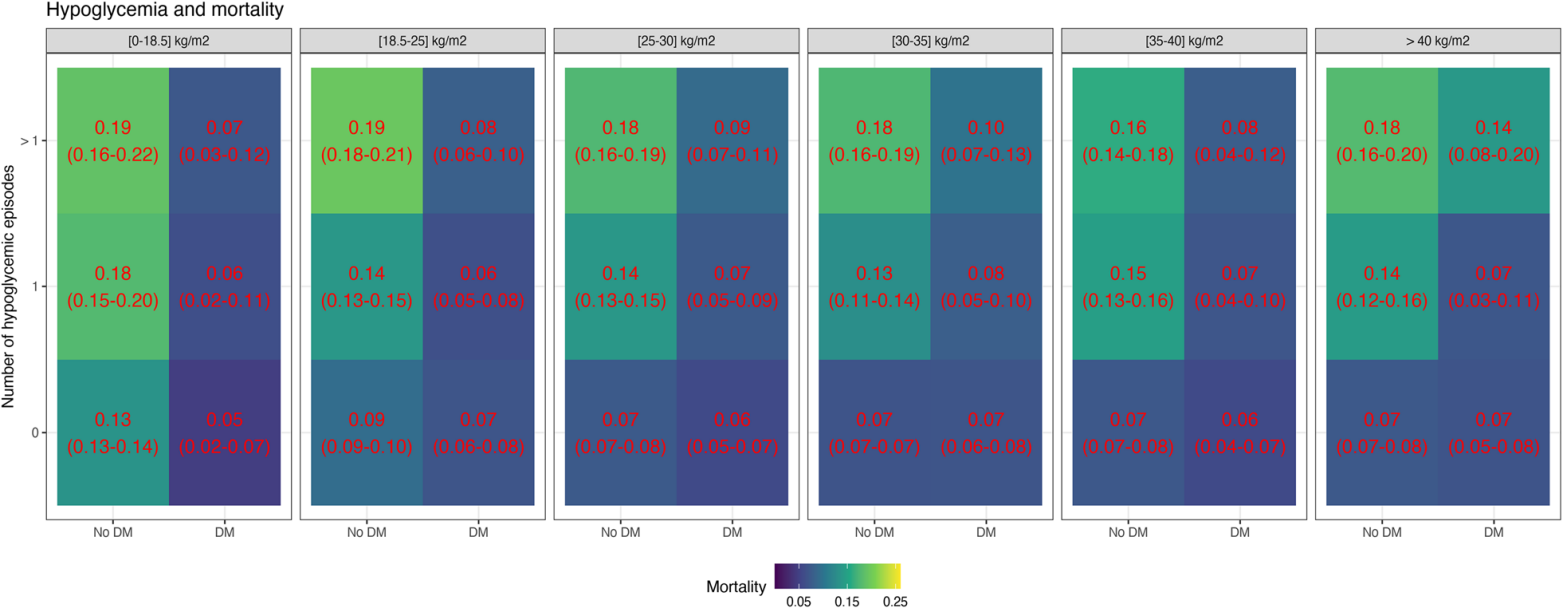
Mortality and hypoglycemia rate association conditional on BMI group and diabetes status. The figure shows the mortality rate in groups with none, a single or multiple episodes of hypoglycemia, conditional on BMI group and diabetes status. For both diabetic and non-diabetic patients, hypoglycemia was associated with increased mortality, for all BMI groups

Increased average glucose levels were associated with increased mortality in each BMI group for non-diabetic patients, although the differences in risk were smaller with higher BMI values (Fig. 6). For diabetic patients, increased glucose levels were associated with reduced mortality (Fig. 6). These associations remained very similar when considering the subgroup of patients who did not develop hypoglycemia during ICU stay (Fig. 7). Similar to average glucose levels, increased glycemic variability was associated with increased mortality in each BMI group for the non-diabetic population, but not for the diabetic population (Fig. 8). Hypoglycemia and its recurrence were associated with increased mortality for both diabetic and non-diabetic groups, and this relationship did not seem strongly modulated by BMI group (Fig. 9).

### Process of care characteristics

Patients with a higher BMI had more frequent glucose monitoring (Additional file 4 panel a, all *p* < 0.001), but we observed the same shape of the hypoglycemic rate curve when considering only patients from the top quartile of glucose measurement frequency across all BMI values (Additional file 4 panel b, all *p* < 0.001). The null-hypothesis that the obesity paradox was not present was rejected in the hypoglycemic cohorts of AUMC and eICU datasets (*p* < 0.001, *p* = 0.024, respectively), whereas it was not rejected in the hypoglycemic cohorts of HiRID and MIMIC-III datasets (*p* = 0.83, *p* = 0.46, respectively, see Additional file 4 panel c). The hypoglycemic load was higher in low BMI patients (Additional file 4 panel e, all *p* < 0.001). It was uncertain whether hypoglycemia was more likely to recur in low BMI patients (Additional file 4 panel f, *p* = 0.01 for AUMC, *p* > 0.05 for others). It was also uncertain whether the lowest glucose level was different according to BMI (Additional file 4 panel d, *p*-values 0.04, 0.07, 0.55 and 0.008, respectively).

Higher BMI patients received higher insulin doses (Additional file 5 panel a, all *p* < 0.001), even when adjusted for patient weight (Additional file 5 panel b, all *p* < 0.001). We observed no consistent association of BMI with dextrose, parenteral and enteral nutrition (Additional file 5 panels c, d, f). Higher BMI patients were less likely to be treated with corticosteroids (all *p* < 0.001), but the association exhibited a U-shape (Additional file 5 panel e). For each BMI group and each database, all process of care characteristics and numbers of patients are additionally reported in Additional file 6.

### Multivariate comparisons

The odds ratios (ORs) of included variables and their confidence intervals (CIs) obtained in the multivariate analysis are presented in Table 2. The maximal GVIF value of the model was 1.55, indicating that multicollinearity did not pose a problem for coefficient estimation. After adjustment, the higher BMI groups 25–30, 30–35, 35–40 kg/m^2^ showed a statistically significant reduction in hypoglycemic rate compared to the normal BMI group (18.5–25 kg/m^2^), with an OR (95% CI reported in parentheses) of 0.72 (0.67–0.77), 0.65 (0.60–0.71) and 0.61 (0.54–0.69), respectively (*p* < 0.001 for all). In addition, the low BMI group showed an increased rate of hypoglycemia compared to the normal weight group (OR 1.6, CI 1.43–1.79) while the > 40 kg/m^2^ group showed a decreased rate of hypoglycemia (OR 0.53, CI 0.46–0.61), although in the sensitivity analysis this finding was not statistically significant (OR 0.83, CI 0.48–1.41), possibly due to low number of morbidly obese patients in the European datasets. The overall findings were consistent when considering the subset of the data with low data missingness (AUMC admissions from 2009 to 2016 and all HiRID admissions, Table 2) in the sensitivity analysis. The findings were also consistent when the diagnosis of diabetes was included as predictor in the US databases (Table 2).

**Table 2:** Estimated odds ratios (ORs) and confidence intervals of important variables in a multivariate logistic model predicting the onset of hypoglycemia (blood glucose ≤ 70 mg/dL) within 6 h

Odds ratio (CI)	Full data	Sensitivity analysis	Diabetes adjusted
BMI [0–18.5] kg/m^2^	1.6 (1.43–1.79)	1.61 (1.28–2.03)	1.58 (1.35–1.86)
*BMI [18.5–25] kg/m*^*2*^	1 (–)	1 (–)	1 (–)
BMI [25–30] kg/m^2^	0.72 (0.67–0.77)	0.74 (0.65–0.85)	0.68 (0.62–0.74)
BMI [30–35] kg/m^2^	0.65 (0.6–0.71)	0.63 (0.52–0.76)	0.59 (0.53–0.66)
BMI [35–40] kg/m^2^	0.61 (0.54–0.69)	0.46 (0.32–0.65)	0.58 (0.5–0.66)
BMI > 40 kg/m^2^	0.53 (0.46–0.61)	0.83 (0.48–1.41)	0.44 (0.38–0.51)
*Blood glucose [70–108] mg/dL*	1 (–)	1 (–)	1 (–)
Blood glucose [108–144] mg/dL	0.3 (0.28–0.32)	0.26 (0.22–0.29)	0.34 (0.31–0.37)
Blood glucose [144–180] mg/dL	0.26 (0.24–0.28)	0.18 (0.15–0.21)	0.34 (0.3–0.38)
Blood glucose > 180 mg/dL	0.29 (0.26–0.32)	0.18 (0.15–0.22)	0.35 (0.31–0.39)
*Insulin* = *0 u/h*	*1* *(–)*	*1* *(–)*	*1* *(–)*
Insulin [0–2.5] u/h	2.18 (2.01–2.35)	3.02 (2.58–3.52)	1.32 (1.17–1.49)
Insulin [2.5–5] u/h	5.07 (4.69–5.48)	6.97 (5.87–8.28)	3.47 (3.12–3.87)
Insulin > 5 u/h	6.41 (5.87–7)	8.76 (7.11–10.79)	4.17 (3.73–4.66)
*Blood lactate [0–2] mmol/L*	1 (–)	1 (–)	1 (–)
Blood lactate [2–5] mmol/L	1.6 (1.48–1.73)	1.45 (1.25–1.7)	1.8 (1.62–2)
Blood lactate > 5 mmol/L	3.24 (2.86–3.68)	3.79 (3.03–4.74)	3.63 (3.03–4.35)
*MAP* ≥ *60 mmHg, no vasopressor therapy*	*1* *(–)*	*1* *(–)*	*1* *(–)*
MAP < 60 mmHg or vasopressor therapy	1.48 (1.39–1.57)	1.41 (1.24–1.6)	1.56 (1.43–1.69)
Parenteral nutrition	0.66 (0.58–0.76)	0.68 (0.52–0.89)	0.75 (0.61–0.92)
Enteral nutrition	0.83 (0.78–0.89)	0.62 (0.55–0.71)	1.11 (1.02–1.21)
Corticosteroids	0.85 (0.77–0.94)	0.81 (0.67–0.98)	0.95 (0.84–1.08)
*Dextrose 10% 0 mL/h*	*1* *(–)*	*1* *(–)*	*1* *(–)*
Dextrose 10% < 25 mL/h	1.06 (0.74–1.52)	0.8 (0.37–1.72)	1.03 (0.69–1.56)
Dextrose 10% > 25 mL/h	2.04 (1.75–2.37)	2 (1.57–2.55)	1.92 (1.51–2.44)
Diabetes	–	–	2.21 (2.04–2.4)
SOFA*	1.07 (1.06–1.08)	1.04 (1.01–1.06)	1.07 (1.06–1.09)
AUMC	1 (–)	1 (–)	–
HiRID	0.43 (0.39–0.48)	0.66 (0.58–0.74)	–
MIMIC–III	0.92 (0.86–0.99)	–	1 (–)
eICU	1.51 (1.38–1.65)	–	2.19 (2–2.4)

Rows corresponding to referent value bins are italicized

ALT, alanine aminotransferase; AST, aspartate aminotransferase; h, hours; IU, international units; MAP, mean arterial pressure; mL, milliliters u/h units per hour

*SOFA score without cardio component

## Discussion

### Key findings

In a study of several large data sets from the USA and Europe, we found that higher BMI patients had higher average glucose levels and more frequent blood glucose measurements, but, despite being more hyperglycemic, also had a reduced risk of death compared to normal or low BMI patients. However, we also found that higher BMI patients had a reduced risk of hypoglycemia and lower glucose variability. We note that these findings were modified by the presence of diabetes. Finally, we confirmed the association of increased BMI and reduced rate of hypoglycemia on multivariate analyses, after including several major time-varying risk factors in logistic regression models.

### Relationship to previous literature

Many of the findings of this study appear to match the findings of several other studies dealing with the obesity paradox [6–11, 13–20]. However, the finding that greater BMI is associated with both hyperglycemia and yet decreased mortality is novel. The same is true of the finding that a higher BMI appears to protect patients from hypoglycemia. In this regard, a higher BMI, like the diagnosis of diabetes [35], appears to remove the nexus between a higher glucose and increased mortality. The finding that the impact of BMI on the association of glycemia with mortality was also modulated  by presence of diabetes is novel and supports the notion that there is an overlapping impact of BMI and diabetes on biochemical and clinical outcomes and that diabetic patients represent a unique population that needs to be considered separately in all assessments of glycemia and its elements and associations [34].

The shape of the hypoglycemia rate curve with respect to BMI values suggests that overweight and mildly obese patients have a particularly lower risk of hypoglycemia. This finding, together with the shape of the risk curve, agrees strongly with those found in a study on severe hypoglycemia during outpatient management of type 2 diabetes [23]. The mechanism by which BMI might affect the risk of hypoglycemia cannot be inferred from our study. One possible explanation relates to insulin resistance, which is known to be associated with higher BMI [36, 37]. As hypoglycemia is an adverse outcome associated with increased mortality [38, 39], the fact that a higher BMI decreases the risk of hypoglycemia might provide a partial explanation for the BMI paradox. However, we acknowledge there are also other possible explanations, or contributing factors, such as the role of fat tissue and adipocytes in absorption and reduction of the inflammatory response [40, 41].

### Implications of study findings

Our findings imply that high BMI patients, like diabetic patients, have decreased mortality despite greater hyperglycemia. Moreover, they imply that higher BMI patients have a lower rate of hypoglycemia, an effect also seen in diabetic patients. Even though there are likely other important factors contributing to the obesity paradox, such as the fact that higher BMI might be an indication of higher lean body mass, our findings imply that a differential impact on glycemia may contribute to the seemingly protective effect of obesity in critical illness [38, 39, 42, 43]. In addition, our findings imply that the association of BMI with mortality is affected by the diagnosis of diabetes, indicating that diabetic patients represent a unique population that reliably modifies the links between most aspects of glycemia with outcome. We acknowledge that some of our findings could also be interpreted as implying underweight is a greater risk for mortality, rather than overweight being protective. However, the changes in risk are in comparison with normal values and can be observed to progress with changes in BMI even within normal BMI values. They also provide a rationale for a precision medicine based approach in relation to glycemic control that would consider the significance and impact of BMI.

### Strengths and limitations

This study has several strengths. It included more than 250,000 ICU patient stays in four large, international cohorts from Europe and the USA. The cohorts were heterogeneous in terms of their surgical and medical admission composition (MIMIC-III predominantly medical, AUMC predominantly surgical), yielding a degree of external validity to the study findings. Moreover, the association of increased BMI and decreased hypoglycemic rate remained significant after adjusting for other known patient and treatment risk factors for hypoglycemia. In multivariate analysis, time-varying risk factors were analyzed only up to the time of hypoglycemia, which attenuated the risk of reverse causality. The consistency of the findings across all four heterogeneous cohorts and the agreement of the findings with existing literature supports the hypothesis that increased BMI has a physiologically protective effect with respect to hypoglycemic rate*.* Therefore, based on this novel finding, BMI can be used as an independent factor in models for hypoglycemia prediction*.*

There are also some limitations to our study. This is an observational study, possibly prone to systematic sampling bias. Moreover, not all patients had a recorded BMI value. However, to address this concern, a sensitivity analysis was performed on a subset of the databases in which the missingness level was low, and the findings remained consistent. The number of patients in the underweight and class II/III obese groups was lower, which is reflected in wider confidence intervals for these groups, limiting the ability to draw definitive conclusions. However, the association still remained that normal BMI patients are at more risk than overweight patients both in terms of mortality and hypoglycemia, and the number of patients in these groups was large. We also acknowledge that BMI is an imperfect anthropometric marker. It does not account for the proportion of lean versus fat body mass, or body fat distribution, both of which might play a role in explaining the observed obesity paradox. Our assessment of the diabetic population provided data to suggest that diabetes might affect the way BMI modulates the link between aspects of glycemia and mortality, but multivariate analyses that would confirm this finding were not performed in this manuscript. Furthermore, such findings were limited to the US databases, where the identification of diabetes was possible. Additionally, HbA1c levels were available only for a subgroup of MIMIC-III patients and based on this data, no definitive conclusions could be made. These limitations are problematic and the data obtained may not be sufficiently robust, because in the MIMIC-III dataset, the mean time between glucose measurements was between 9 and 10 h, reflecting a monitoring frequency of < 3 times a day. The detection rate of dysglycemia in both diabetic and non-diabetic patients could be impacted by such a low measurement rate. Finally, the use of oral hypoglycemic agents was not considered in our analyses. The AUMC database was the only database reporting the use of oral hypoglycemic agents. It reported such use in 0.5% of total patient stay days, indicating they were not widely used in this dataset.

## Conclusion

Our findings provide novel evidence that, compared with a normal BMI, a BMI above the normative value is an independent predictive factor for reduced risk of hypoglycemia in diabetic and non-diabetic patients, while a BMI below the normal value predicts increased risk. These findings suggest the need to consider BMI when assessing the significance of hypoglycemia as a predictor of mortality and when estimating the risk of hypoglycemia in critically ill patients. Additionally, our study also confirms the inverse relationship of increasing BMI values and mortality, a relationship which appears to occur despite greater hyperglycemia (a known predictor of mortality) with increasing BMI, but which also appears to be modified by the presence of diabetes. In their aggregate, our findings imply that future studies of glycemia in critically ill patients should control for the impact of both diabetes and BMI.

## Supplementary Information

**Additional file 1**. Comparison of characteristics of patients with a recorded BMI against patients with a missing BMI. A tabular summary of demographic characteristics (age, gender) and important outcomes (mortality, hypoglycemia, length of stay, SOFA score) for patients with a recorded BMI and patients with a missing BMI.

**Additional file 2**. Missingness of key physiological parameters across BMI groups in the first day of ICU admission (figure). A visual summary of missingness of creatinine, lactate, bilirubin, platelets, PaO2/FiO2 ratio, Mean Arterial Pressure, GCS and glucose.

**Additional file 3**. Missingness of key physiological parameters across BMI groups in the first day of ICU admission (table). A tabular summary of missingness of creatinine, lactate, bilirubin, platelets, PaO2/FiO2 ratio, Mean Arterial Pressure, GCS and glucose.

**Additional file 4**. Process of care analyses. **a** Higher BMI patients are more frequently monitored for blood glucose levels; **b** The association of decreased rate of hypoglycemia and high BMI also appears for patients who are in the top quartile of glucose measurement frequency; **c** The association of mortality and BMI for hypoglycemic patients only; **d** The severity of hypoglycemia across BMI groups; **e** The hypoglycemic load across BMI groups; **f** The recurrence of hypoglycemia across BMI groups.

**Additional file 5**. Association of BMI and key treatment variables related to glycemic control. A visual summary of the association of BMI groups with insulin rate, weight-normalized insulin rate, parenteral nutrition duration, enteral nutrition duration, corticosteroids duration and dextrose infusion rate, across all four databases. The proportion of patients treated in each database is reported in parentheses in each panel after the database name.

**Additional file 6**. Process of care characteristics. A tabular summary of process of care characteristics, including time-weight average glucose, mean time between consecutive blood glucose measurements, mean maximal insulin, mean weight-normalized maximal insulin, mean duration of parenteral nutrition, mean duration of enteral nutrition, mean duration of corticosteroid treatment and mean dextrose infusion rate.

## Data Availability

The datasets supporting the conclusions of this article are available upon request. The AUMC database is available through the website of Amsterdam Medical Data Science (https://amsterdammedicaldatascience.nl/), whereas HiRID, MIMIC-III and eICU databases are available via PhysioNet (https://physionet.org/).

## Code availability

All the code used is publicly available at https://github.com/eth-mds/bmi.
