# Supplementary material for: The obesity paradox and hypoglycemia in critically ill patients
Source: Crit Care. 2021 Nov 1;25:378. doi: 10.1186/s13054-021-03795-z (PMC8559391; doi:10.1186/s13054-021-03795-z)
Supplement: Supplementary file 1 — Additional file 1. Comparison of characteristics of patients with a recorded BMI against patients with a missing BMI. A tabular summary of demographic characteristics (age, gender) and important outcomes (mortality, hypoglycemia, length of stay, SOFA score) for patients with a recorded BMI and patients with a missing BMI. [file 13054_2021_3795_MOESM1_ESM.docx]

**Additional file 1.** Comparison of characteristics of patients with a recorded BMI against patients with a missing BMI.

| Variable | Reported | BMI recorded | BMI missing |
| --- | --- | --- | --- |
| Cohort size | n | 259177 | 51385 |
| Admission type  - *Medical*  *- Surgical*  *- Other* | % | 71  26  3 | 70  28  2 |
| Age (years) | Median  (IQR) | 66  (54-77) | 65  (53-76) |
| Mortality | % | 9 | 11 |
| Hypoglycemia | % | 14 | 14 |
| ICU LOS | Median  (IQR) | 1.67  (0.9-3.25) | 1.25  (0.68-2.56) |
| Hospital LOS (days) | Median  (IQR) | 5.92  (3.11-10.72) | 5.9  (3.04-10.97) |
| Gender (male) | % | 57 | 54 |
| SOFA score  - *Cardiovascular*  *- CNS*  *- Coagulation*  *- Hepatic*  *- Renal*  *- Respiratory*  Total | Median (IQR) | 1 (1-1)  0 (0-1)  0 (0-1)  0 (0-0)  0 (0-1)  2 (0-3)  4 (2-6) | 1 (0-1)  0 (0-1)  0 (0-1)  0 (0-1)  0 (0-1)  1 (0-2)  3 (1-5) |

CNS central nervous system; IQR interquartile range; LOS length of stay; NR not reported; SOFA Sequential Organ Failure Assessment, evaluated at 24 hours into ICU stay.
