# Supplementary figures and images for: The obesity paradox and hypoglycemia in critically ill patients
Source: Crit Care. 2021 Nov 1;25:378. doi: 10.1186/s13054-021-03795-z (PMC8559391; doi:10.1186/s13054-021-03795-z)

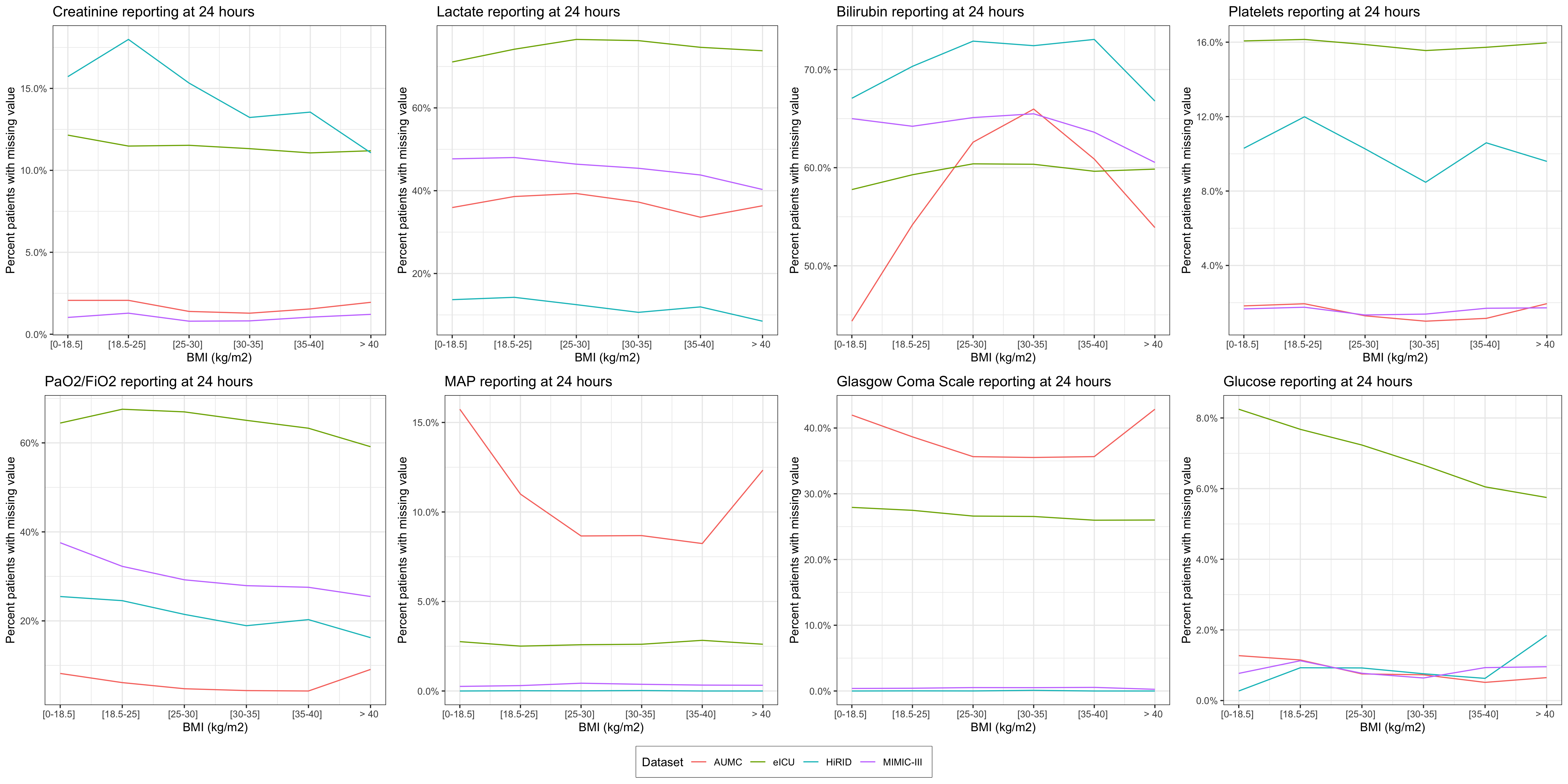

Supplement: Supplementary file 2 — Additional file 2. Missingness of key physiological parameters across BMI groups in the first day of ICU admission (figure). A visual summary of missingness of creatinine, lactate, bilirubin, platelets, PaO2/FiO2 ratio, Mean Arterial Pressure, GCS and glucose. [file 13054_2021_3795_MOESM2_ESM.tiff]

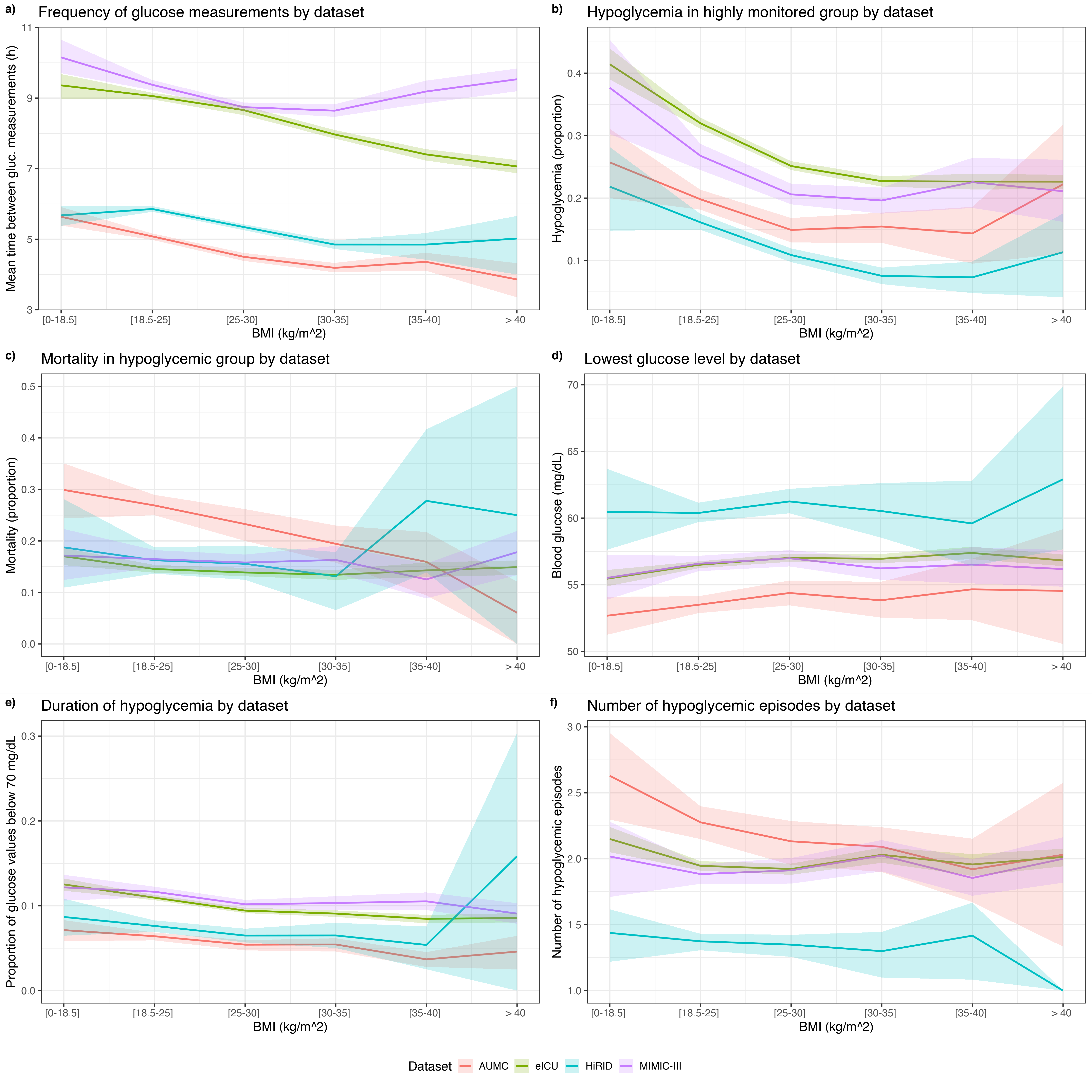

Supplement: Supplementary file 4 — Additional file 4. Process of care analyses. a Higher BMI patients are more frequently monitored for blood glucose levels; b The association of decreased rate of hypoglycemia and high BMI also appears for patients who are in the top quartile of glucose measurement frequency; c The association of mortality and BMI for hypoglycemic patients only; d The severity of hypoglycemia across BMI groups; e The hypoglycemic load across BMI groups; f The recurrence of hypoglycemia across BMI groups. [file 13054_2021_3795_MOESM4_ESM.tiff]

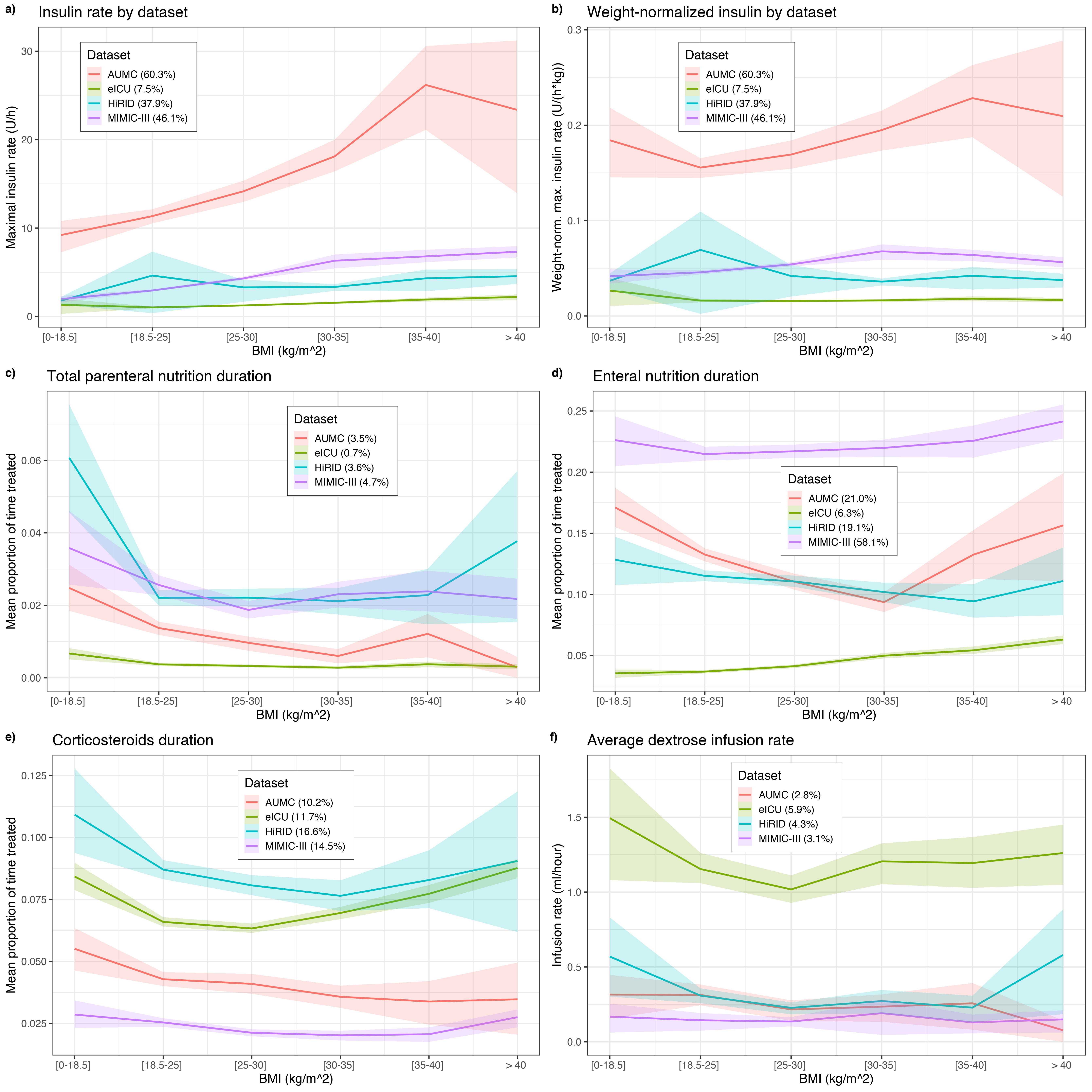

Supplement: Supplementary file 5 — Additional file 5. Association of BMI and key treatment variables related to glycemic control. A visual summary of the association of BMI groups with insulin rate, weight-normalized insulin rate, parenteral nutrition duration, enteral nutrition duration, corticosteroids duration and dextrose infusion rate, across all four databases. The proportion of patients treated in each database is reported in parentheses in each panel after the database name. [file 13054_2021_3795_MOESM5_ESM.tiff]
