# Supplementary material for: The obesity paradox and hypoglycemia in critically ill patients
Source: Crit Care. 2021 Nov 1;25:378. doi: 10.1186/s13054-021-03795-z (PMC8559391; doi:10.1186/s13054-021-03795-z)
Supplement: Supplementary file 3 — Additional file 3. Missingness of key physiological parameters across BMI groups in the first day of ICU admission (table). A tabular summary of missingness of creatinine, lactate, bilirubin, platelets, PaO2/FiO2 ratio, Mean Arterial Pressure, GCS and glucose. [file 13054_2021_3795_MOESM3_ESM.docx]

**Additional file 3: Table S2.** Missingness of key variables at 24 hours into ICU stay. The numbers indicate the percent of patients who do not have a recorded value for the respective feature in the first 24 hours of ICU stay, for each dataset and BMI group.

| Dataset | BMI group (kg/m^2^) | Creatinine | Lactate | Bilirubin | Platelets | PaO2/FiO2 | MAP | GCS | Glucose |
| --- | --- | --- | --- | --- | --- | --- | --- | --- | --- |
| *AUMC* | [0-18.5] | 2 | 36 | 44 | 2 | 8 | 16 | 42 | 1 |
|  | [18.5-25] | 2 | 39 | 54 | 2 | 6 | 11 | 39 | 1 |
|  | [25-30] | 1 | 39 | 63 | 1 | 5 | 9 | 36 | 1 |
|  | [30-35] | 1 | 37 | 66 | 1 | 4 | 9 | 36 | 1 |
|  | [35-40] | 2 | 34 | 61 | 1 | 4 | 8 | 36 | 1 |
|  | > 40 | 2 | 36 | 54 | 2 | 9 | 12 | 43 | 1 |
|  |  |  |  |  |  |  |  |  |  |
| *HiRID* | [0-18.5] | 16 | 14 | 67 | 10 | 25 | 0 | 0 | 0 |
|  | [18.5-25] | 18 | 14 | 70 | 12 | 25 | 0 | 0 | 1 |
|  | [25-30] | 15 | 12 | 73 | 10 | 21 | 0 | 0 | 1 |
|  | [30-35] | 13 | 11 | 72 | 8 | 19 | 0 | 0 | 1 |
|  | [35-40] | 14 | 12 | 73 | 11 | 20 | 0 | 0 | 1 |
|  | > 40 | 11 | 8 | 67 | 10 | 16 | 0 | 0 | 2 |
|  |  |  |  |  |  |  |  |  |  |
| *MIMIC-III* | [0-18.5] | 1 | 48 | 65 | 2 | 38 | 0 | 0 | 1 |
|  | [18.5-25] | 1 | 48 | 64 | 2 | 32 | 0 | 0 | 1 |
|  | [25-30] | 1 | 46 | 65 | 1 | 29 | 0 | 1 | 1 |
|  | [30-35] | 1 | 45 | 65 | 1 | 28 | 0 | 1 | 1 |
|  | [35-40] | 1 | 44 | 64 | 2 | 28 | 0 | 1 | 1 |
|  | > 40 | 1 | 40 | 61 | 2 | 25 | 0 | 0 | 1 |
|  |  |  |  |  |  |  |  |  |  |
| *eICU* | [0-18.5] | 12 | 71 | 58 | 16 | 64 | 3 | 28 | 8 |
|  | [18.5-25] | 11 | 74 | 59 | 16 | 68 | 3 | 27 | 8 |
|  | [25-30] | 12 | 77 | 60 | 16 | 67 | 3 | 27 | 7 |
|  | [30-35] | 11 | 76 | 60 | 16 | 65 | 3 | 27 | 7 |
|  | [35-40] | 11 | 75 | 60 | 16 | 63 | 3 | 26 | 6 |
|  | > 40 | 11 | 74 | 60 | 16 | 59 | 3 | 26 | 6 |

FiO2 fraction of inspired oxygen; GCS Glasgow Coma Scale score; MAP mean arterial pressure; PaO2 partial arterial O2 pressure.
