# Supplementary material for: The obesity paradox and hypoglycemia in critically ill patients
Source: Crit Care. 2021 Nov 1;25:378. doi: 10.1186/s13054-021-03795-z (PMC8559391; doi:10.1186/s13054-021-03795-z)
Supplement: Supplementary file 6 — Additional file 6. Process of care characteristics. A tabular summary of process of care characteristics, including time-weight average glucose, mean time between consecutive blood glucose measurements, mean maximal insulin, mean weight-normalized maximal insulin, mean duration of parenteral nutrition, mean duration of enteral nutrition, mean duration of corticosteroid treatment and mean dextrose infusion rate. [file 13054_2021_3795_MOESM6_ESM.docx]

**Additional file 5.** Number of patients and process of care characteristics (average time-weighted glucose, glucose monitoring frequency, maximal insulin, maximal insulin normalized by weight, parenteral and enteral nutrition, corticosteroids, dextrose) for each BMI group.

| Dataset | BMI group (kg/m^2^) | | No. of patients | | Mean time-weighted avg glucose (mg/dL) | | Mean time between gluc. meas. (hours) | | Mean Maximal Insulin (U/h) | | Mean Weight-norm. Maximal Insulin (U/(h*kg)) | | | Mean duration of parenteral nutrition (h) | Mean duration of enteral nutrition (h) | Mean duration of corticosteroid treatment (h) | Mean dextrose infusion rate (mL/h) |
| --- | --- | --- | --- | --- | --- | --- | --- | --- | --- | --- | --- | --- | --- | --- | --- | --- | --- |
| *AUMC* | [0-18.5] | | 1433 | | 130.91 | | 5.64 | | 9.21 | | 0.18 | | 0.02 | | 0.17 | 0.06 | 0.32 |
|  | [18.5-25] | | 10484 | | 135.54 | | 5.08 | | 11.35 | | 0.16 | | 0.01 | | 0.13 | 0.04 | 0.31 |
|  | [25-30] | | 5557 | | 138.21 | | 4.50 | | 14.16 | | 0.17 | | 0.01 | | 0.11 | 0.04 | 0.22 |
|  | [30-35] | | 3123 | | 141.84 | | 4.19 | | 18.10 | | 0.19 | | 0.01 | | 0.09 | 0.04 | 0.24 |
|  | [35-40] | | 851 | | 143.90 | | 4.36 | | 26.19 | | 0.23 | | 0.01 | | 0.13 | 0.03 | 0.26 |
|  | > 40 | | 176 | | 142.22 | | 3.86 | | 23.37 | | 0.21 | | 0.00 | | 0.16 | 0.03 | 0.08 |
|  | |  | |  | |  | |  | |  | |  |  |  |  |  |  |
| *HiRID* | [0-18.5] | | 740 | | 133.42 | | 5.68 | | 1.80 | | 0.04 | | 0.06 | | 0.13 | 0.11 | 0.57 |
|  | [18.5-25] | | 13677 | | 139.23 | | 5.86 | | 4.63 | | 0.07 | | 0.02 | | 0.12 | 0.09 | 0.31 |
|  | [25-30] | | 11589 | | 145.80 | | 5.35 | | 3.29 | | 0.04 | | 0.02 | | 0.11 | 0.08 | 0.23 |
|  | [30-35] | | 3957 | | 151.38 | | 4.85 | | 3.34 | | 0.04 | | 0.02 | | 0.10 | 0.08 | 0.27 |
|  | [35-40] | | 1114 | | 154.54 | | 4.85 | | 4.32 | | 0.04 | | 0.02 | | 0.09 | 0.08 | 0.23 |
|  | > 40 | | 271 | | 153.20 | | 5.02 | | 4.55 | | 0.04 | | 0.04 | | 0.11 | 0.09 | 0.58 |
|  | |  | |  | |  | |  | |  | |  |  |  |  |  |  |
| *MIMIC-III* | [0-18.5] | | 780 | | 131.36 | | 10.15 | | 1.97 | | 0.04 | | 0.04 | | 0.23 | 0.03 | 0.17 |
|  | [18.5-25] | | 7628 | | 131.16 | | 9.38 | | 2.94 | | 0.05 | | 0.03 | | 0.21 | 0.03 | 0.14 |
|  | [25-30] | | 8286 | | 133.46 | | 8.74 | | 4.30 | | 0.05 | | 0.02 | | 0.22 | 0.02 | 0.14 |
|  | [30-35] | | 4539 | | 138.69 | | 8.64 | | 6.31 | | 0.07 | | 0.02 | | 0.22 | 0.02 | 0.19 |
|  | [35-40] | | 1824 | | 139.75 | | 9.19 | | 6.80 | | 0.06 | | 0.02 | | 0.23 | 0.02 | 0.13 |
|  | > 40 | | 1566 | | 140.11 | | 9.53 | | 7.32 | | 0.06 | | 0.02 | | 0.24 | 0.03 | 0.15 |
|  | |  | |  | |  | |  | |  | |  |  |  |  |  |  |
| *eICU* | [0-18.5] | | 8018 | | 134.27 | | 9.36 | | 1.92 | | 0.04 | | 0.01 | | 0 | 0.11 | 2.14 |
|  | [18.5-25] | | 53648 | | 136.04 | | 9.06 | | 2.35 | | 0.04 | | 0.01 | | 0 | 0.10 | 2.15 |
|  | [25-30] | | 52973 | | 139.73 | | 8.66 | | 3.04 | | 0.04 | | 0.01 | | 0 | 0.08 | 1.89 |
|  | [30-35] | | 33378 | | 145.31 | | 7.97 | | 3.23 | | 0.03 | | 0.01 | | 0 | 0.10 | 2.79 |
|  | [35-40] | | 16802 | | 150.86 | | 7.41 | | 3.87 | | 0.04 | | 0.02 | | 0 | 0.12 | 2.23 |
|  | > 40 | | 16763 | | 154.95 | | 7.06 | | 8.42 | | 0.06 | | 0.02 | | 0 | 0.13 | 1.97 |
